# O9.8. STRESS AND COGNITIVE FUNCTION AMONG INDIVIDUALS AT CLINICAL HIGH-RISK FOR PSYCHOSIS: FINDINGS FROM THE NAPLS COHORT

**DOI:** 10.1093/schbul/sby015.252

**Published:** 2018-04-01

**Authors:** Alexis Cullen, Kristin S Cadenhead, Jean Addington, Carrie E Bearden, Tyrone Cannon, Barbara A Cornblatt, Daniel Mathalon, Thomas H McGlashan, Diana O Perkins, Larry Seidman, William S Stone, Ming Tsuang, Scott W Woods, Elaine F Walker

**Affiliations:** 1 Institute of Psychiatry, Psychology & Neuroscience, King’s College London; 2 University of California, San Diego; 3 University of Calgary; 4 University of California, Los Angeles; 5 Yale University; 6 Zucker Hillside Hospital; 7 University of California, San Francisco; 8 University of North Carolina, Chapel Hill; 9 Harvard Medical School; 10 Harvard Medical School, Beth Israel Deaconess Medical Center; 11 Emory University

## Abstract

**Background:**

Accumulated evidence from non-human animal studies suggests that the prominent deficits in memory and executive function that characterise individuals with psychosis may, at least in part, be due to the effects of stress on the brain regions that support these functions. However, studies of patients with established psychosis have yielded inconsistent findings with regards to the relationship between stress and cognition, and research in high-risk populations is notably lacking. Utilising data from the North American Prodrome Longitudinal Study 2 (NAPLS 2), we aimed to further elucidate the relationship between stress (daily stressors, life events, and childhood trauma) and cognitive function in clinical high-risk (CHR) individuals and healthy controls (HC). We additionally explored the role of potential mediators [hypothalamic-pituitary-adrenal (HPA) axis function] and moderators (group status, sex, family history of illness).

**Methods:**

The sample comprised 885 participants (CHR=646; HC=239) who completed measures of stress and cognitive function at the NAPLS 2 baseline assessment. Stress measures included the Daily Stress Inventory and a modified version of the Psychiatric Epidemiology Research Interview Life Events Scale, both of which provided continuous measures of stress exposure (number of events) and distress (subjective feelings of distress). Participants were also interviewed using the Childhood Trauma and Abuse Scale to determine any exposure to childhood trauma (abuse, neglect, and bullying occurring prior to age 16 years). Basal HPA axis activity was determined via salivary cortisol samples obtained at the baseline assessment and standardised scores from selected subtests from the Measurement and Treatment Research to Improve Cognition in Schizophrenia (MATRICS) were used to derive two cognitive domain scores (memory and executive function). To examine relationships between stress and cognitive domain scores, linear regression analyses were performed on standardised variables.

**Results:**

Daily stressor exposure, daily stressor distress, and life event exposure exhibited negative quadratic (i.e., inverted U-shaped) associations with both memory and executive function (P < 0.01 for all). In contrast, the reverse pattern (i.e., a negative linear relationship and a positive quadratic relationship) was shown in the model for life event distress and memory domain scores (P < 0.01) whilst trauma history showed only a trend-level association with poorer memory performance (P = 0.084). These relationships, which did not differ across CHR and healthy control groups, were largely unchanged after adjusting for demographic factors and salivary cortisol. Exploratory analyses suggested that trauma exposure and a family history of psychosis may moderate the relationship between daily stressors/life events and cognitive function.

**Discussion:**

In this large sample of predominately CHR individuals, we observed that the association between stress and cognition is complex and differs across stressor types. The negative quadratic associations that we observed for daily stressor exposure, daily stressor distress, and life event exposure imply that whist lower levels of stress may facilitate memory and executive function, there may be a negative impact on cognition when these stressors become more frequent and distressing. Interventions aiming to minimise stress exposure and promote effective coping strategies might feasibly improve cognition in CHR individuals.

